# Effects of ketamine on circadian rhythm and synaptic homeostasis in patients with treatment‐resistant depression: A protocol for mechanistic studies of its rapid and sustained antidepressant actions in humans

**DOI:** 10.1002/brb3.1423

**Published:** 2019-10-15

**Authors:** Chuanjun Zhuo, Hongjun Tian, Gongying Li, Min Chen, Deguo Jiang, Xiaodong Lin, Yong Xu, Wenqiang Wang

**Affiliations:** ^1^ School of Mental Health Jining Medical University Jining China; ^2^ Psychiatric‐Neuroimaging‐Genetics Laboratory Wenzhou Seventh People's Hospital Wenzhou China; ^3^ Psychiatric‐Neuroimaging‐Genetics‐Comorbidity Laboratory (PNGC_Lab) Tianjin Mental Health Centre Mental Health Teaching Hospital of Tianjin Medical University Tianjin Anding Hospital Tianjin China; ^4^ Department of Psychiatry School of Basic Medical Science Tianjin Medical University Tianjin China; ^5^ Department of Psychiatry First Hospital/First Clinical Medical College of Shanxi Medical University Taiyuan China; ^6^ MDT Center for Cognitive Impairment and Sleep Disorders First Hospital of Shanxi Medical University Taiyuan China; ^7^ Co‐collaboration Laboratory of China and Canada Xiamen Xianyue Hospital and University of Alberta Xiamen China

**Keywords:** circadian rhythm, depression, functional connectivity, functional magnetic resonance imaging, ketamine, major depressive disorder, synaptic homeostasis, treatment‐resistant depression

## Abstract

**Background:**

The breakthrough discovery has been made that a single dose of ketamine, an N‐methyl‐D‐aspartate receptor antagonist, achieves rapid and sustained (~7 days) antidepressant activity in patients with major depressive disorder (MDD). This discovery has ushered in an exciting era of research and brought new hope for patients with MDD. However, the mechanisms underlying the specific antidepressant actions of ketamine in humans remain to be elucidated.

**Objectives:**

This study protocol was designed to test the main hypothesis that ketamine could rapidly reverse depression‐ and stress‐associated synaptic loss and deficits in resting‐state functional connectivity and that this action could be affected by circadian rhythm, in patients with treatment‐resistant depression.

**Methods/Study Design:**

In this clinical study, adults (aged 18–65 years) with treatment‐resistant depression will be randomized to intravenous administration of placebo (control group) or ketamine (0.5 mg/kg body weight) at 11 a.m. (daytime group), or 6 p.m. (nighttime group) for 24 weeks. The primary outcome will be the change from baseline to 24 weeks in the total Montgomery‐Asberg Depression Rating Scale score. Brain imaging, sleep, and genetic studies, including functional magnetic resonance imaging, positron emission tomography, polysomnography, and genetic analyses, will be performed to examine whether and how ketamine can rapidly reverse deficits in synaptic function and to identify objective markers for the assessment of ketamine infusion therapy for treatment‐resistant depression.

**Conclusions:**

This clinical study protocol is the first, to our knowledge, to describe the prospective testing of the hypothesis that daytime and nighttime administrations of ketamine would have different antidepressant effects. The brain imaging, sleep, and genetic findings from patients with treatment‐resistant depression are expected to shed new light on the mechanisms of ketamine and its interaction with target sites in the brain, which can be used for objective evaluation of the efficacy of ketamine.

## Graphical abstract

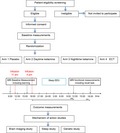

## 1 INTRODUCTION

### 1.1 Major depressive disorder and current medications

Major depressive disorder (MDD), referred to simply as depression, affects approximately 17% of the global population, and its incidence appears to be increasing (Kessler, 2012; Murray et al., 2013). Depression is predicted to become one of the main causes of disability worldwide (Kessler, 2012; Murray et al., 2013). Current mediations for MDD, notably monoamine reuptake inhibitors of monoamine neurotransmitters (e.g., serotonin, norepinephrine, dopamine), usually require weeks to months from treatment initiation to achieve full clinical response; this time lag, as well as the moderate effectiveness or lack of efficacy of such drugs, has led to an increased risk of suicide and even suicide occurrence among depressed patients. Moreover, as many as one‐third of patients with MDD respond inadequately to two or more monoamine reuptake inhibitors in combination and are considered to have treatment‐resistant depression (Trivedi et al., 2006). Thus, the development of better care delivery to patients with MDD, especially those with treatment‐resistant depression, is urgently needed.

### 1.2 Ketamine as a breakthrough discovery due to its rapid and sustained antidepressant effects

In recent decades, a breakthrough discovery was made for ketamine, an N‐methyl‐D‐aspartate (NMDA) receptor antagonist and dissociative anesthetic, which has been proven by multiple lines of evidence to possess rapid and sustained (~1 week) antidepressant actions (Berman et al., 2000; Sinner & Graf, 2008; Zarate et al., 2006). In addition to having a mild dissociative effect, a single low dose of ketamine (0.5 mg/kg, i.v. slow infusion) has been demonstrated to have robust efficacy for treatment‐resistant depression relative to conventional antidepressants (Duman, Shinohara, Fogaca, & Hare, 2019). The U.S. Food and Drug Administration recently approved the use of esketamine, the S enantiomer of ketamine, for the management of treatment‐refractory MDD in adult patients at imminent risk of suicide, and designated esketamine nasal spray as a breakthrough therapy.

Ketamine is a mixture with two equal enantiomers: R‐ketamine and S‐ketamine (Hashimoto, 2019). Previous studies have shown that each enantiomer has its own advantages and disadvantages (Chaki, 2017; Hashimoto, 2019; Kohrs & Durieux, 1998; Yang et al., 2015). For instance, R‐ketamine exerts more prolonged antidepressant activities than does S‐ketamine in rodent models of depression (Chaki, 2017; Hashimoto, 2019; Kohrs & Durieux, 1998; Yang et al., 2015). S‐ketamine has been proven to possess greater affinity for the NMDA receptor than does R‐ketamine and thus is considered to be a more potent and active stereoisomer of racemic ketamine (Chaki, 2017; Kohrs & Durieux, 1998). In this study, we will administer a racemic mixture of ketamine containing equal amounts of R‐ketamine and S‐ketamine to human subjects.

### 1.3 Potential mechanisms underlying the rapid and sustained antidepressant effects of ketamine

The exciting findings mentioned above have prompted great interest among scientists in the study of the mechanisms by which ketamine exerts its rapid and sustained antidepressant actions. Although the exact mechanisms remain unclear, progress has been made recently with animal (predominantly rodent) models of depression.

#### 1.3.1 Ketamine rapidly reverses synaptic deficits in animal models of depression

One potential mechanism of action is that ketamine could reverse depression, chronic stress‐related synaptic loss, or deficits in synaptic connectivity through a burst of glutamate, which produces rapid synaptic actions that underlie antidepressant behavioral responses (Autry et al., 2011; Duman et al., 2019). The possibility that synaptogenic effects have a role in the antidepressant actions of ketamine is also supported by evidence that stress and depression are associated with decreased synapse number and atrophy of the cortical and limbic brain regions (Autry et al., 2011; Li et al., 2010; Moghaddam, Adams, Verma, & Daly, 1997). Several studies of this potential mechanism in rodent models of depression have shown that ketamine rapidly increases levels of several important synaptic proteins, including synapsin 1, GluA1, PSD95, and mPFC within ≤2 hr following administration (Giuliano et al., 2011; Li et al., 2010; Maeng et al., 2008; Zanos et al., 2016), which is consistent with its prompt antidepressant actions (Berman et al., 2000; Zarate et al., 2006). Notably, rapid enhancement of some synaptic proteins, in particular GluA1, an important member of the excitatory neurotransmitter glutamate receptor family and a main subunit of the alpha‐amino‐3‐hydroxy‐5‐methyl‐4‐isoxazole propionate receptor and ligand‐activated cation channels leads to increases in the number and function of synapses (Giuliano et al., 2011; Li et al., 2010; Liu & Aghajanian, 2008). In animal models of depression and chronic unpredictable stress (CUS, a core symptom of depression), NMDA glutamate receptor antagonists rapidly reversed chronic stress‐induced synaptic deficits (Duman et al., 2019; Giuliano et al., 2011; McEwen et al., 2015; McEwen & Morrison, 2013). Interestingly, compared with the rapid reversal of CUS‐caused anhedonic behavior and synaptic deficits after a single dose of ketamine, traditional medications for depression, such as MRIs of monoamine neurotransmitters, reversed CUS‐related anhedonic behavior as long as 3 weeks after administration (Giuliano et al., 2011). These findings support the differences in efficacy between typical antidepressants and ketamine.

Although the results obtained from rodent models of depression or with the core symptom of depression are exciting, whether ketamine can reverse atrophy and synaptic loss, thereby targeting the underlying neurobiology of depression, in humans, remains to be determined. Brain imaging studies, such as functional magnetic resonance imaging (fMRI) studies, are needed to test this hypothesis in patients with depression; such research should include clinical studies investigating whether ketamine could reverse the volumetric changes observed in the hippocampus and prefrontal cortex (PFC) in patients diagnosed with depression. *UCB‐J*, a new synaptic positron emission tomography (PET) ligand, has become available to bind to the synaptic vesicle glycoprotein 2A (SV2A), an essential membrane glycoprotein expressed in virtually all synapses (Chen et al., 2018). Using UCB‐J radiolabeled with ^11^C (^11^C‐UCB‐J) in PET imaging, Chen and colleagues (Chen et al., 2018) investigated alterations in synaptic density in patients with Alzheimer disease (AD). Their results suggested that SV2A was a suitable target for the in vivo examination of synaptic density in human subjects. Taking advantage of this new approach, we plan to study the effects of ketamine on synaptic density in human studies.

#### 1.3.2 Ketamine improves disrupted circadian and sleep rhythms

Circadian rhythms are correlated primarily with the sleep–wake cycle (Masri & Sassone‐Corsi, 2013), and depression is recognized as a mental illness that is correlated strongly with disrupted circadian and sleep rhythms. A recent study revealed a strong correlation between depression and poor quality of sleep, involving the impairment of functional connectivity in multiple regions of the brain, including the lateral orbital frontal cortex, dorsolateral PFC, anterior/posterior cingulate cortex, insula, hippocampus, amygdala nuclei, temporal lobe, and precuneus (Cheng, Rolls, Ruan, & Feng, 2018). In another study, electrophysiology revealed significant changes in auditory evoked potentials in patients with MDD (Goldstein et al., 2012). Moreover, slow‐wave sleep deprivation therapy effectively improved the core symptoms of depression (Landsness, Goldstein, Peterson, Tononi, & Benca, 2011). Recently, a subgroup of patients with MDD was found to have abnormal circadian processes, including interruptions in sleep, hormone secretions, mood, and temperature, all of which were modulated by circadian clock genes (Bunney et al., 2015). Interestingly, some studies also have shown that circadian rhythms return to normal as depression symptoms remit (Avery, Shah, Eder, & Wildschiodtz, 1999; Hasler, Buysse, Kupfer, & Germain, 2010; Souetre et al., 1988; Troxel et al., 2012). A recent study from the University of California at Irvine involved transcriptome profiling to identify genes and pathways in relation to ketamine‐associated alterations in circadian and sleep rhythms in mice (Orozco‐Solis et al., 2017). Ketamine treatment led to a rapid and significant reduction in immobility compared with the control saline treatment (Orozco‐Solis et al., 2017), consistent with the findings of several previous studies (Autry et al., 2011; Hines, Schmitt, Hines, Moss, & Haydon, 2013; Lopez‐Rodriguez, Kim, & Poland, 2004; Scheuing, Chiu, Liao, & Chuang, 2015). Further comparative transcriptomics analyses revealed that several key rhythmic genes (e.g., Ciart, Per2, Npas4, Dbp, and Rorb) were differentially expressed in the brain in response to ketamine treatment in mice (Orozco‐Solis et al., 2017). Several studies have demonstrated that ketamine enhanced rapid eye movement (REM) sleep and significantly increased levels of brain‐derived neurotrophic factor (BDNF), a synaptic protein correlated strongly with slow‐wave activity (SWA), to improve BDNF‐mediated synaptic plasticity and depressive symptoms (Ballard et al., 2016; Duncan et al., 2017; Evans et al., 2018; Monteggia & Zarate, 2015; Zarate & Machado‐Vieira, 2017). These findings suggest the involvement of circadian and sleep rhythms in the rapid, antidepressant response to ketamine.

Initial scientific evidence for the abnormal expression of circadian clock genes in the brain in patients with MDD came from a microarray study, which showed that circadian rhythms in as many as six brain areas were significantly altered, with the most disrupted brain area being the anterior cingulate cortex (ACC), in patients with MDD relative to control individuals (Li et al., 2013). The ACC is well recognized as a main component of an extended neural network, with a role in the regulation of mood. A growing body of findings has implicated the ACC as an important area of the brain associated with depression (Drevets, Savitz, & Trimble, 2008). Functional brain imaging studies also have shown that ketamine significantly increased ACC activation (Salvadore et al., 2009). Ketamine may reset key circadian and sleep rhythms, thereby exerting sustained antidepressant effects. However, whether nighttime administration of ketamine could improve its efficacy in patients with MDD remains unknown. The potential attribution of ketamine's sustained antidepressant effect to the modulation of circadian and sleep rhythms warrants further study.

## 2 STUDY OBJECTIVES

This study protocol is designed to examine the mechanisms underlying the rapid and sustained antidepressant actions of ketamine in humans and to test the central hypothesis that ketamine could rapidly reverse depression‐ and stress‐associated synaptic deficits in patients with treatment‐resistant depression, using brain imaging. In addition, this clinical study will be, to our knowledge, the first to prospectively assess the hypothesis that day and night administrations of ketamine would result in different antidepressant actions. We plan to test the main hypothesis and, thereby, to meet the study objective, by pursuing the following specific aims and conducting the proposed studies in patients with treatment‐resistant or treatment‐refractory depression. The long‐term goal of our ongoing research program is to gain neurobiological knowledge of how depression is formed, and how it can be cured through the translation of scientific findings into new efficacious therapeutic approaches.

Specific aim #1: To determine the optimal timing of ketamine infusion therapy for treatment‐resistant depression.

Working hypothesis 1: We hypothesize that day and night administrations of ketamine will have different effects on the efficacy of ketamine for treatment‐resistant depression.
Working hypothesis 2: We hypothesize that ketamine administered at night will work better than ketamine administered during the day.

Specific aim #2: To determine whether and how ketamine can rapidly reverse deficits in synaptic function, particularly volumetric abnormalities in the PFC and hippocampus, in patients with treatment‐resistant depression.

Working hypothesis 1: We hypothesize that ketamine will reverse chronic stress‐ and depression‐related deficits in synaptic connectivity in the PFC and hippocampus in patients with treatment‐resistant depression.
Working hypothesis 2: We hypothesize that ketamine will recover chronic stress‐ and depression‐related volumetric abnormalities in the PFC and hippocampus in patients with treatment‐resistant depression.
Working hypothesis 3: We hypothesize that ketamine treatment will markedly reverse deficits in synaptic density in the PFC and hippocampus compared with baseline, as measured by the percentage of SV2A‐specific binding on PET scans.
Working hypothesis 4: We hypothesize that patients who receive ketamine at night will show better synapse restoration in the PFC and hippocampus than will those who receive ketamine during the day, as assessed by the percentage of SV2A‐specific binding on PET scans.

Specific aim #3: To identify and establish objective markers for the assessment of ketamine infusion therapy for treatment‐resistant depression using brain imaging studies.

Working hypothesis 1: We hypothesize that brain MRI studies of patients with treatment‐resistant depression will reveal the interaction of ketamine with its target sites in the brain, which could be linked to the drug's pharmacological effects and used for objective evaluation of the efficacy of ketamine.
Working hypothesis 2: We hypothesize that in vivo synapse assessment using ^11^C‐UCB‐J‐PET imaging will enable the direct measurement of synaptic density as a potential new objective marker or outcome measure of the efficacy of ketamine.
Working hypothesis 3: We hypothesize that MRI‐ and PET‐observed alterations will be linked, enabling the establishment of an “MRI–PET bridge” with tremendous potential as more useful approach in the next 10 years.

This study is innovative and highly significant within the field. Completion of the proposed research is expected to (a) shed new light on the mechanisms underlying ketamine's rapid and sustained antidepressant actions in patients with treatment‐resistant depression, (b) offer a more efficacious approach for ketamine infusion therapy for treatment‐resistant depression, and (c) provide suggestions for objective markers or measures for evaluation of the efficacy of ketamine for treatment‐resistant depression, or other clinical trials of therapies targeting synapse restoration.

## 3 METHODS AND STUDY DESIGN

**Figure 1:**
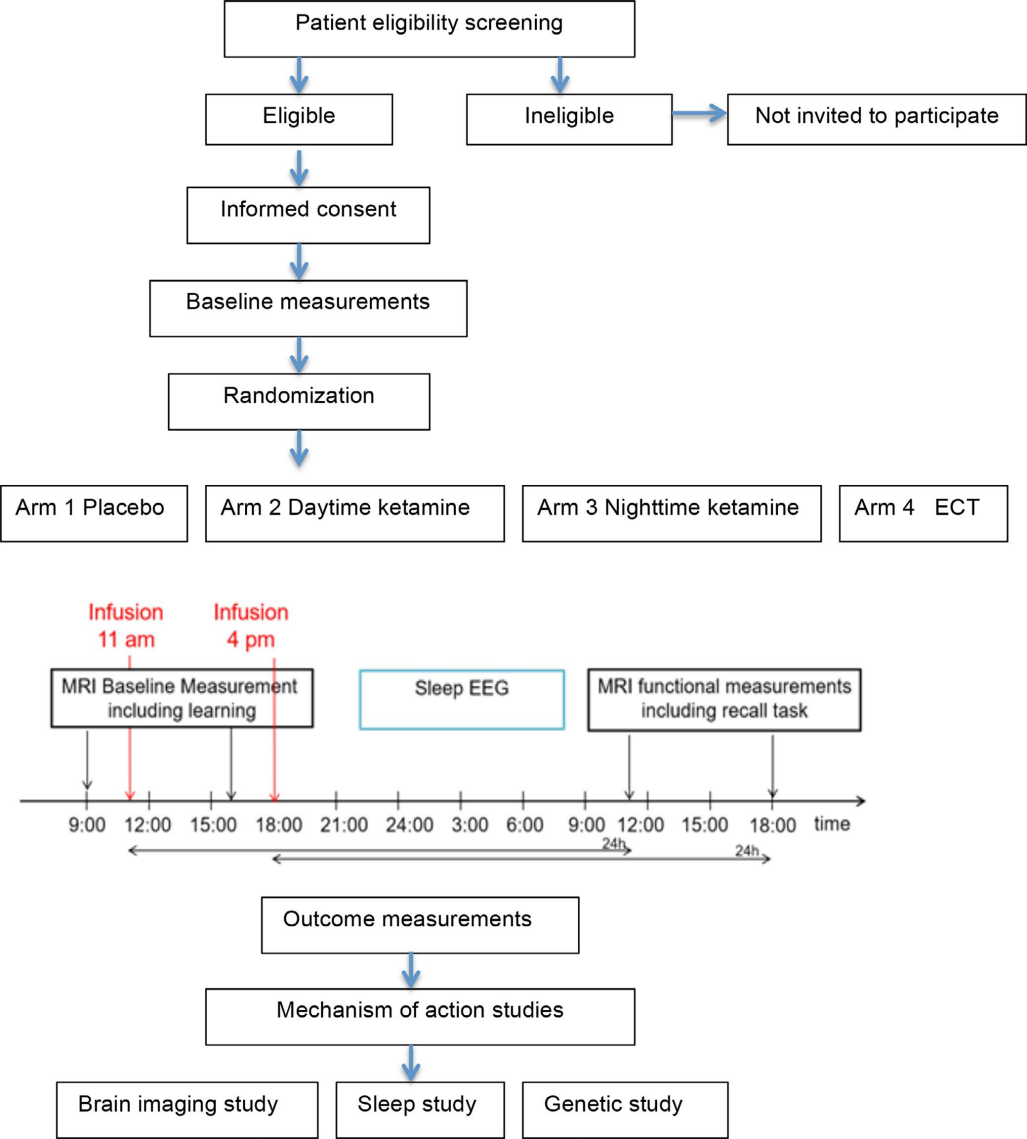
Flowchart of the study design

This study protocol is designed to test the main hypothesis that ketamine could rapidly reverse depression‐ and stress‐associated synaptic losses or deficits in resting‐state functional connectivity (rsFC) and that this action could be affected by circadian rhythms, in patients with treatment‐resistant depression. The design of this study is illustrated in Figure 1.

### 3.1 Human subjects

Potential patients will be enrolled from the Tianjin Mental Health Center affiliated with Tianjin Medical University in Tianjin, a coastal metropolis in northern China with approximately 13,000,000 residents. Eligible patients will (a) be aged 18–65 years to minimize potential older age‐related confounding effects, such as AD and cognitive impairment; (b) meet the Diagnostic and Statistical Manual of Mental Disorders fifth edition criteria for recurrent MDD without psychotic features, which will be confirmed using the Mini International Neuropsychiatric Interview (Sheehan et al., 1998; Singh et al., 2016); (c) have inadequate responses to at least two antidepressant medications, with at least one antidepressant failure to treat current depressive episodes, as evaluated by medical histories, the Massachusetts General Hospital Antidepressant Response Questionnaire, and Montgomery‐Asberg Depression Rating Scale (MADRS) score ≥21 at baseline (Chandler, Iosifescu, Pollack, Targum, & Fava, 2010; Ng et al., 2019; Singh et al., 2016); (d) be willing to participate and fully cooperate in this study; and (e) provide written informed consent prior to study participation. Patients with the following conditions will be considered to be ineligible and will be excluded from the study: (a) severe systemic disease; (b) epilepsy, claustrophobia, primary obsessive–compulsive disorder, anorexia nervosa, bulimia nervosa, or posttraumatic stress disorder; (c) medical history or current diagnosis of a psychotic disorder; (d) medical history or current diagnosis of mental retardation, bipolar disorder, mood disorder with postpartum onset, borderline personality disorder, or somatoform disorder; (e) hypertension or vascular disease, including aneurysm, vascular malformation, thrombosis, and neoplasm; (f) unfitness for MRI, including the presence of a magnetic implant; (g) unfitness for PET, including allergy to PET tracers; (h) substance abuse; (i) use of medicine for a sleep disorder; (j) medical history of nonresponsiveness of depressive symptoms to ketamine; (k) clinically significant suicidal or homicidal ideation or imminent risk of harm; and (l) current pregnancy or breastfeeding status. Independent World‐Class Services for Drug Development and Clinical Trial Success (SAFER) raters from Massachusetts General Hospital will be used to verify that all randomized patients meet the SAFER criteria, have treatment‐resistant MDD based on the Antidepressant Treatment Response Questionnaire, and manifest the required depression severity.

The study protocol will be submitted to and reviewed by the Ethics Committee of the Mental Health Center affiliated with Tianjin Medical University (Tianjin, China). The proposed study will be conducted in accordance with the Declaration of Helsinki and in consistency with good clinical practices. All enrolled patients will undergo preliminary examination, during which they will be informed of the study background and procedures, as well as the potential risks and benefits. With full awareness and prior to study initiation, study participants will provide written informed consent.

Participants will be able to withdraw from the study without specifying a reason during the treatment period. Supervising physicians may decide to terminate a patients' participation if his/her condition deteriorates.

### 3.2 Randomization and treatment

R/S‐ketamine hydrochloride, sold under the brand name Ketalar (Pfizer Pharmaceuticals), will be used as the study drug. A total of 600 adult patients with treatment‐resistant depression will be enrolled in this study. Randomization and stratification of the study patients will be carried out using the Randomization in Treatment Arms software (Evident). The patients will be assigned to the following three groups (200 per group): placebo, daytime ketamine administration, and nighttime ketamine administration. Patients in the ketamine groups will be given ketamine infusions at a dose of 0.5 mg/kg (R/S‐ketamine hydrochloride diluted in saline, administered slowly by i.v. pump for >40 min) in the morning and at night, respectively (Berman et al., 2000; Zarate et al., 2006); patients in the placebo group will receive matching saline infusions (Figure 1). These patients will receive two infusions per week for 24 weeks (Singh et al., 2016). Treatments will be administered in private rooms at the Mental Health Teaching Hospital affiliated with Tianjin Medical University.

### 3.3 Clinical assessment and monitoring

Participants' blood pressure, heart rate, blood oxygen levels, and other clinical parameters will be measured and recorded before the initiation of ketamine infusion and at 10, 20, 30, 40, 80, 120, and 240 min thereafter. Electrical activity in the brain, mainly sleep parameters, will be monitored for 24 hr using an electroencephalogram (EEG).

### 3.4 Polysomnographic monitoring and scoring of sleep and associated events

Polysomnography (PSG) will be performed using a Nicolet v32 device (Natus Medical Incorporated). Audio–video recording, along with continuous recording of thermopressure air flow, blood oxygen saturation, and diaphragm movement, and EEG, electro‐oculography, and electromyography, will be performed. Before PSG, all patients will avoid the consumption of caffeine‐containing beverages, and the protocol will be explained to them protocol to relieve nervousness. PSG will register shifts between REM and nonrapid eye movement (NREM) sleep phases (divided further into deep and light sleep phases), identified by well‐trained technicians following the American Academy of Sleep Medicine's (AASM's) Manual for the Scoring of Sleep and Associated Events version 2.5, updated and released in April 2018 (American Academy of Sleep Medicine; The AASM Manual for the Scoring of Sleep and Associated Events: American Academy of Sleep Medicine; https://aasm.org/resources/pdf/scoring-manual-preface.pdf). The main parameters recorded will be the total sleep time, sleep latency, REM latency, sleep efficiency, phase ratio, and the apnea–hypopnea index.

The specific phases of sleep will be identified by continuous recording systems at a rate of 30 frames/s. According to the AASM manual (2018, version 2.5), the wake phase is identified when the occipital α rhythm occupies more than 50%, or one of the following features is present even without a recognizable α component: (a) eye blinking at 0.5–2 Hz; (b) regular eyeball movement; or (c) irregular conjugate REM with normal or slightly higher mentalis tension. The NREM stage 1 phase is identified when a vertex sharp wave or low‐voltage (4–7 Hz) mixed‐frequency dominates with α synchrony in closed eyes. NREM stage 2 is recognized with single or multiple arousal‐irrelevant K complex wave/sleep spinal waves, with <20% of SWA in the current frame. NREM stage 3 is defined by more than 20% of SWA in the current frame. The REM sleep phase is deduced from the simultaneous occurrence of low‐voltage mixed‐frequency EEG signals, lower mentalis tension, and irregular conjugate REM.

### 3.5 Magnetic resonance imaging and analysis

All study patients will undergo two fMRI examinations for three‐dimensional (3D) magnetization‐prepared rapid gradient‐echo (MPRAGE) imaging, rsFC assessment, and diffusion‐weighted imaging (DWI) using an advanced GE Signa HDx 3.0T MR system (GE Healthcare) in accordance with the recommendation of the Human Connectome Project for mapping neural connections of the human brain (http://www.neuroscienceblueprint.nih.gov/connectome/). Two hours before the initiation of ketamine infusion, patients in the daytime ketamine administration group will be scheduled for baseline fMRI examination at 9 a.m., and those in the nighttime ketamine administration group will undergo baseline fMRI examinations at 4 p.m. Twenty‐four hours after baseline fMRI, repeat fMRI examinations will be performed for patients in these two groups (Figure 1).

Functional magnetic resonance imaging examinations will be performed using the following parameters. For T1‐weighted 3D MPRAGE structural imaging of the whole brain, 128‐layer sagittal scanning will be carried out for 4 min using the following parameters: 1.33 mm thickness, 0‐mm interval, 256 × 192 in‐layer resolution, 2,530‐ms repetition time (TR), 3.39‐ms echo time (TE), 7° flip angle (FA), and 256 × 256 mm^2^ field of view (FOV). For rsFC assessment, 33‐layer axial imaging will be undertaken for 10 min with the following parameters: 3 mm thickness, 0‐mm interval, 64 × 64 in‐layer resolution, 2,000‐ms TR, 30‐ms TE, 90° FA, and 220 × 220‐mm^2^ FOV. For the digital trunk interface (DTI) module, the following parameters will be used: 70 layers with 2 mm thickness, 0‐mm interval, 7,100/61‐ms TR/TE, 256 × 256‐mm^2^ FOV, and 128 × 128 matrix. The gradient direction will be set to 64 (*b* = 1,000, *b*0 image = 10, NEX = 1). For the diffusion kurtosis imaging (DKI) module, a 2D multislice single‐shot spin‐echo echo‐planar‐imaging sequence will be used with 48 layers of 3 mm thickness, 0‐mm interval, 5,800/77‐ms TR/TE, 90° FA, 256 × 256 mm^2^ FOV, and 128 × 128 matrix. The gradient directions will be set to 25 (*b* = 1,000) and 25 (*b* = 2,000), with *b*0 image = 10 and NEX = 1. For magnetic resonance spectrometry, a single voxel spectrum will be located in the ACC; the TE will be set to 2,000 ms.

In MRI examination, sagittal 3D T1‐weighted images will be acquired using the following parameters: 188 sagittal slices, 1 mm slice thickness, no gap, 8.2‐ms TR, 3.2‐ms TE, 450‐ms inversion time (TI), 12° FA, 256 × 256 mm^2^ FOV, and 256 × 256 matrix. Resting‐state fMRI data will be acquired using a single‐short gradient‐echo echo‐planar‐imaging sequence with the following parameters: 32 interleaved transverse slices, 4 mm slice thickness, 0.5‐mm gap, 2,000/45‐ms TR/TE, 90° FA, 220 × 220 mm^2^ FOV, 64 × 64 matrix, and 180 volumes. For analysis of the brain white matter, a single‐shot spin‐echo‐planar‐imaging sequence will be used with three diffusion weightings (*b* = 1,000, 1,500, and 2,000 s/mm^2^), 30 noncollinear directions, and five *b* = 0 s/mm^2^ volumes (13,000‐ms TR, 86.1‐ms TE, 1.88 × 1.88 × 2.50‐mm^3^ voxel size). The diffusion gradient length (*δ*) and spacing (Δ) will be held constant (*δ*/Δ = 35.1/44.7 ms). Raw images will be denoised, corrected for Gibbs ringing, and corrected for eddy currents and motion using the *eddy* tool in the FMRIB Software Library (version 6.0; Analysis Group, FMRIB). DTI and DKI parameters will be calculated using weighted linear least‐squares estimation (https://github.com/NYU-DiffusionMRI/Diffusion-Kurtosis-Imaging).

### 3.6 PET imaging and analysis

For PET imaging, 40 patients will be randomly selected from the daytime and nighttime ketamine administration groups (20 per group) to undergo PET scans using the ^11^C‐UCB‐J PET ligand at baseline and upon completion of the 24‐week treatment period. This subset of patients will be included in consideration of the difficulties involved in brain PET, including poor patient compliance and high cost (Rausch et al., 2017; Thompson et al., 2016). In brief, PET imaging will be performed using a high‐resolution research tomography (GE Health Care) with a reconstructed image resolution of nearly 3 mm, as described previously (Finnema et al., 2018; Nabulsi et al., 2016). These patients will also undergo T1‐weighted MRI imaging in a 3‐T whole‐body scanner (GE HealthCare) at the same timepoints for coregistration with the PET images.

### 3.7 Blood sampling and molecular biological studies

Peripheral blood samples (5 ml) will be collected from all patients at baseline and upon treatment completion for the quantification of ketamine levels and molecular biological studies. The blood samples will be centrifuged at 5,000 *g* for 10 min, and the serum samples will be stored at −80°C in a freezer. An enzyme‐linked immunosorbent assay kit (Sigma‐Aldrich) will be used for the measurement of serum BDNF levels on a SpectraMax M5 microplate reader (Molecular Devices) at a wavelength of 450 nm. All samples will be measured in triplicate. Genomic DNA will be extracted from the blood samples and used for subsequent genetic polymorphism genotyping with two genes of interest (Homer1 and BDNF), performed at the molecular core laboratory of Tianjin Medical University. Researchers blinded to the clinical data will perform the genotyping of Homer1 at rs7713917 (the A allele indicates a higher risk of dysregulation of cognitive and motivational processes through effects on prefrontal activity during anticipation of reward), rs2290639 (the AA homozygote was associated significantly with suicide attempts in Chinese patients in Hong Kong), and rs60029291 (the T allele was associated with MDD and suicide attempts in Chinese patients), as described previously (Serchov et al., 2015).

### 3.8 Sample size determination and power analysis

The effective sample size has been estimated using G‐Power analysis according to the G * Power 3.1 manual, released in March 2017 (http://www.gpower.hhu.de/en.html). Assuming a 15% dropout rate in each group and to observe significant effects with an *α* value of 0.05 and statistical power of 0.8, a total of 800 patients with treatment‐resistant depression will be enrolled in the randomized, double‐blinded control study.

### 3.9 Outcome measures

The primary outcome measure will be the change from baseline to the treatment completion in the total MADRS score (0–6, normal or absence of symptoms; 7–19, mild depression; 20–34, moderate depression; >34, severe depression; Cunningham, Wernroth, Knorring, Berglund, & Ekselius, 2011; Herrmann, Black, Lawrence, Szekely, & Szalai, 1998; Muller‐Thomsen, Arlt, Mann, Mass, & Ganzer, 2005; Williams & Kobak, 2008). The MADRS will be used to assess the effects of ketamine in the three groups, specifically with the following 10 items, which are used widely for the measurement of depression severity: (a) apparent sadness, (b) reported sadness, (c) inner tension, (d) reduced sleep, (e) reduced appetite, (f) concentration difficulty, (g) lassitude, (h) inability to feel, (i) pessimistic thoughts, and (j) suicidal thoughts (Cunningham et al., 2011; Herrmann et al., 1998; Muller‐Thomsen et al., 2005; Williams & Kobak, 2008).

Patients will undergo objective cognitive testing at baseline and upon treatment completion. The Montreal Cognitive Assessment will serve as the primary cognitive measure (Nasreddine et al., 2005). The North American Adult Reading Test‐35 (Uttl, 2002) will be used to estimate intellectual function. The revised Hopkins Verbal Learning Test, Controlled Oral Word Association Test, and Stroop Color and Word Test will also be administered at baseline and at the end of treatment. Percentages of SV2A‐specific binding for synapse measurement on PET images will be calculated and compared between the two ketamine groups, and between baseline and completion of the 24‐week treatment period in each ketamine group.

### 3.10 Side effects and safety

The occurrence of adverse events and side effects, including memory complaints reported by patients and recorded by physicians, will also be evaluated. Ketamine has been reported to be associated with neurocognitive impairments, manifesting mainly as memory recall problems (Murrough et al., 2013). Patients' neurocognitive function will also be assessed using a comprehensive battery including the estimated premorbid intelligence quotient (IQ), current IQ, and tests from the MATRICS Consensus Cognitive Battery, as described previously (Murrough et al., 2013). Data on patients' self‐reported memory complaints will be collected using the Squires Memory Complaint Questionnaire, Global Self‐Evaluation of Memory, and Patient‐Rated Inventory of Side Effects. Data on physician‐reported adverse events and side effects will be collected using the Brief Psychiatric Rating Scale psychotic subscale and the Clinician‐Administered Dissociative State Scale.

Upon the development of serous medical complications, including but not limited to suicidal ideation, cardiovascular toxicity, and infusion‐related side effects, the participants will be advised to withdraw from the study and will receive prompt care in the emergency room.

### 3.11 Data handling and statistical methods

Baseline demographic information and clinical characteristics of the enrolled patients will be compared among groups using one‐way analysis of variance (ANOVA) or the Kruskal–Wallis *H* test, as appropriate, for continuous variables, and the chi‐squared test or Fisher's exact test, as appropriate, for nominal variables. The effects of different treatments on MADRS scores will be examined using one‐way ANOVA, followed by post hoc testing of ketamine versus placebo and daytime versus nighttime ketamine administration. A multiple linear regression analysis of estimated conditional treatment effects will be conducted for effect measure modification.

The IBM SPSS version 20.0 software (IBM Corp.) will be utilized for statistical analyses. *p* values <.05 will be taken to indicate significant differences between groups. The statistical software and methods used may change if newer software becomes available.

## 4 DISCUSSION

A single dose of ketamine is sufficient to achieve rapid and relatively sustained antidepressant effects and to reduce the risk of suicide, in patients with MDD. Previous studies have revealed that ketamine is involved in circadian and sleep rhythms, as well as in reversing synaptic deficits in animal models of depression. Clinical examination of the neurobiological mechanisms underlying the specific actions of ketamine in human subjects is required. Based on our long‐standing interest in the study of MDD and on our preliminary findings, we present this protocol for investigation of the mechanisms by which ketamine exerts its unique antidepressant actions in patients with refractory depression. The anticipated findings and their implications, as well as potential limitations, are discussed below.

### 4.1 Anticipated results and implications

This study will mainly test the overarching hypothesis that ketamine will rapidly reverse depression‐ and stress‐associated synaptic deficits and reset disrupted circadian and sleep rhythms in patients with treatment‐resistant depression. These two mechanisms have different neurological pathways, and potential interactive effects have not been explored to date. Furthermore, we will test the efficacy of different ketamine interventions (daytime and nighttime administration), using saline as a control. We anticipate that the findings will shed new light on the mechanism through which, at least in part, ketamine exerts its prompt and long‐lasting actions in patients with treatment‐resistant depression, improve ketamine infusion therapy for MDD, and provide suggestions for potential objective markers of the efficacy of ketamine infusion therapy for treatment‐resistant depression. Thus, we anticipate that the findings of this study will advance our understanding of the mechanisms underlying the antidepressant effects of ketamine. Although the results from this research project will stem from the specific context of ketamine, data obtained through the direct comparative analysis of different treatment approaches (daytime vs. nighttime ketamine administration) will have a number of implications for better clinical practice in the management of treatment‐resistant depression, which may affect patient choice and health insurance policy. Additionally, the findings hold promise to guide the development of a more efficacious approach for ketamine infusion therapy and thus may eventually improve care for patients with MDD, particularly those with treatment‐resistant depression. Such a new approach could be easily adopted in clinical practice. Notably, the study of SV2A‐specific binding to synapses using ^11^C‐UCB‐J‐PET will allow us to examine synapses directly in vivo, and we expect that it will lead to the identification of a new objective marker or outcome measure of the efficacy of ketamine for treatment‐resistant depression, or of other clinical trials of therapies targeting synapse restoration. The combined use of PET and MRI may reveal correlations between MRI‐ and PET‐observed alterations, or the “MRI–PET bridge.” This “bridge” is an important part of our study, and we anticipate that it will have significant clinical implications as more useful approach in the next 10 years.

### 4.2 Limitations of this study protocol

Despite the obvious strengths of this study, some potential limitations of our study protocol should be noted. First, we recognize that this entire pilot study will be conducted at a single center and that selection bias may be a weakness. In addition, our hospital‐based enrollment method could affect our findings. Second, although the sample size was calculated to generate sufficient power for the assessment of differences in intervention efficacy among study groups, it may not be large enough to enable analysis of subgroups, such as those defined by sex and genetic variants. Third, although we do not anticipate this to be the case, it is possible that differences in certain patient characteristics could affect the outcomes. Another weakness of this protocol could be that we will not be able to stratify randomization by sex.

## 5 CONCLUSIONS

Despite the potential limitations of the proposed clinical pilot study, the anticipated findings have important implications related to a better understanding of the mechanisms and improvement of the efficacy of ketamine. We believe that the limitations will be addressed in future studies. More importantly, this study is expected to form the basis for future multicenter, large‐scale clinical trials.

## CONFLICT OF INTEREST

All authors declare that they have no competing interest.

## AUTHORS' CONTRIBUTIONS

All authors made substantial, direct, and intellectual contributions to the work and approved it for publication.

## Data Availability

All data are available in the main text.
